# Molecular Imaging of Opioid and Dopamine Systems: Insights Into the Pharmacogenetics of Opioid Use Disorders

**DOI:** 10.3389/fpsyt.2019.00626

**Published:** 2019-09-18

**Authors:** Jamie A. Burns, Danielle S. Kroll, Dana E. Feldman, Christopher Kure Liu, Peter Manza, Corinde E. Wiers, Nora D. Volkow, Gene-Jack Wang

**Affiliations:** ^1^National Institute on Alcohol Abuse and Alcoholism, Bethesda, MD, United States; ^2^National Institute on Drug Abuse, Bethesda, MD, United States

**Keywords:** opioid use disorder, neuroimaging, genetics, positron emission tomography, PET, polymorphism, opioid receptors, dopamine receptors

## Abstract

Opioid use in the United States has steadily risen since the 1990s, along with staggering increases in addiction and overdose fatalities. With this surge in prescription and illicit opioid abuse, it is paramount to understand the genetic risk factors and neuropsychological effects of opioid use disorder (OUD). Polymorphisms disrupting the opioid and dopamine systems have been associated with increased risk for developing substance use disorders. Molecular imaging studies have revealed how these polymorphisms impact the brain and contribute to cognitive and behavioral differences across individuals. Here, we review the current molecular imaging literature to assess how genetic variations in the opioid and dopamine systems affect function in the brain’s reward, cognition, and stress pathways, potentially resulting in vulnerabilities to OUD. Continued research of the functional consequences of genetic variants and corresponding alterations in neural mechanisms will inform prevention and treatment of OUD.

## Introduction

Opioid use in the United States has steadily risen since the late 1990s, along with staggering increases in overdose fatalities (1). The use of illicit opioids such as heroin and fentanyl has increased dramatically, contributing to opioid-related morbidity and mortality (2). With approximately 115 Americans dying each day from an opioid overdose, this epidemic is now considered a public health emergency (3). The surge in prescription and illicit opioid abuse necessitates further investigation into the genetic risk factors and neuropsychological effects of opioid use disorder (OUD).

The roles of the opioid and dopamine (DA) systems in substance use disorders (SUDs) are well recognized (4). Drug reward and incentive salience develop during the acute effects of drug-taking and correspond to changes in opioid and DA signaling in the basal ganglia (5). Incentive salience is defined by the association of previously neutral stimuli with drug use, which promotes compulsive drug-seeking (4). Stress responses associated with withdrawal involve decreased DA signaling along reward pathways, increased dynorphin-mediated kappa opioid (KOP) receptor signaling, and increased corticotropin-releasing factor (CRF) signaling in the amygdala (4). These same principles apply to OUD. For example, Wang et al. (6) used positron-emission tomography (PET) imaging with [^11^C]raclopride to demonstrate lower dopamine receptor 2 (D2R) and 3 (D3R) availability in the striatum of opioid-dependent patients compared to controls. Another [^11^C]raclopride PET study found low striatal D2/3 receptor availability and low presynaptic DA in OUD patients compared to controls (7), which has also been found for other SUDs including cocaine, alcohol, methamphetamine, and cannabis [reviewed in Refs. (8, 9)]. Low D2R levels have also been associated with sleep deprivation (10–12) and lower socioeconomic status (13, 14). These factors may contribute to lower D2R availability found in SUDs, particularly since SUDs and sleep deprivation are highly comorbid (15). Other preclinical studies have found dynorphin-mediated KOP receptor signaling inhibits dopaminergic signaling and modulates aversive emotional states that maintain drug dependence (16–18). Based on these studies, both the opioid and DA signaling systems are implicated in OUD.

However, there are opposing views on these systems’ involvement in addiction. For example, there are studies that report no disruption of D2R in OUD, including no difference in baseline D2R availability in methadone-maintained OUD patients compared to controls (19). Moreover, PET studies of opioid-dependent patients on medications for OUD (MOUD) found no increase in striatal DA release in response to opioid administration (19, 20). Studies of other SUDs also present slight inconsistencies in their effects on the dopamine system. Imaging studies in individuals with alcohol use disorder (AUD) have reported marked reductions in dopamine release and in striatal D2R, and most preclinical studies have documented significant reductions in dopamine neuronal firing and tonic dopamine release (9, 21–27). However, studies in rodents have also reported dynamic changes in dopamine release with increases and decreases in accumbens at various days post alcohol withdrawal (28). The discrepancies in the preclinical studies are likely to reflect in part time at which the measurements were made (early versus late withdrawal) as well as the alcohol models used (active versus passive administration). Thus, further research is required to understand the complex relationship between opioid and DA systems in SUDs.

While it has long been postulated that genetics influence an individual’s susceptibility to addiction, there has been little success in pinpointing genes with well-defined, causal roles in SUDs (29). Nevertheless, OUD is highly heritable, with an estimated 50% genetic contribution (30–32). The use of candidate gene studies and genome-wide association studies has revealed several polymorphisms that reliably associate with SUDs; however, addiction is a polygenic disease with complex genetic interactions and therefore individual polymorphisms will likely only account for a fraction of the total genetic risk for OUD (33–35). Polymorphisms in the opioid signaling system have been associated with addiction, as well as addiction treatment response (29). For example, several studies have identified a single nucleotide polymorphism (SNP) in the *OPRM1* gene that associates with improved response to naltrexone treatment in individuals with AUD (36–39). Other *OPRM1* SNPs may also play a role in nicotine dependence and treatment response (40–42). Additionally, genetic variations in the DA system have been linked to various SUDs as DA modulates reward and aversion pathways central to addiction (29, 43). For example, polymorphisms in the genes coding for dopamine 1 receptor (D1R) and D2R are associated with OUD, cocaine use disorder (CUD), and AUD (6, 22, 44). In addition, polymorphisms in the gene *DAT1*, which codes for dopamine transporters (DAT), have been associated with CUD and AUD (45–47). In line with this, reduced striatal DAT availability has been associated with OUD (48–53) and DAT availability has been associated with various other SUDs (51, 54–62).

In this review, we compiled findings related to the genetics of the opioid and DA systems and corresponding changes in brain and behavior as evidenced by PET neuroimaging. Functional and structural magnetic resonance imaging (MRI) is another useful tool in examining altered neural circuits in individuals with SUDs, as well as in polymorphism carriers. However, we will limit the scope to molecular imaging as the literature on MRI in OUD was recently reviewed (63–66). Integrating genetics with regional changes in receptor binding may help uncover circuits relevant for the pathophysiology of OUD, and thereby inform precision-based prevention and treatment.

## The Opioid Receptor System

### OPRM1

#### *OPRM1* Background

**Figure 1:**
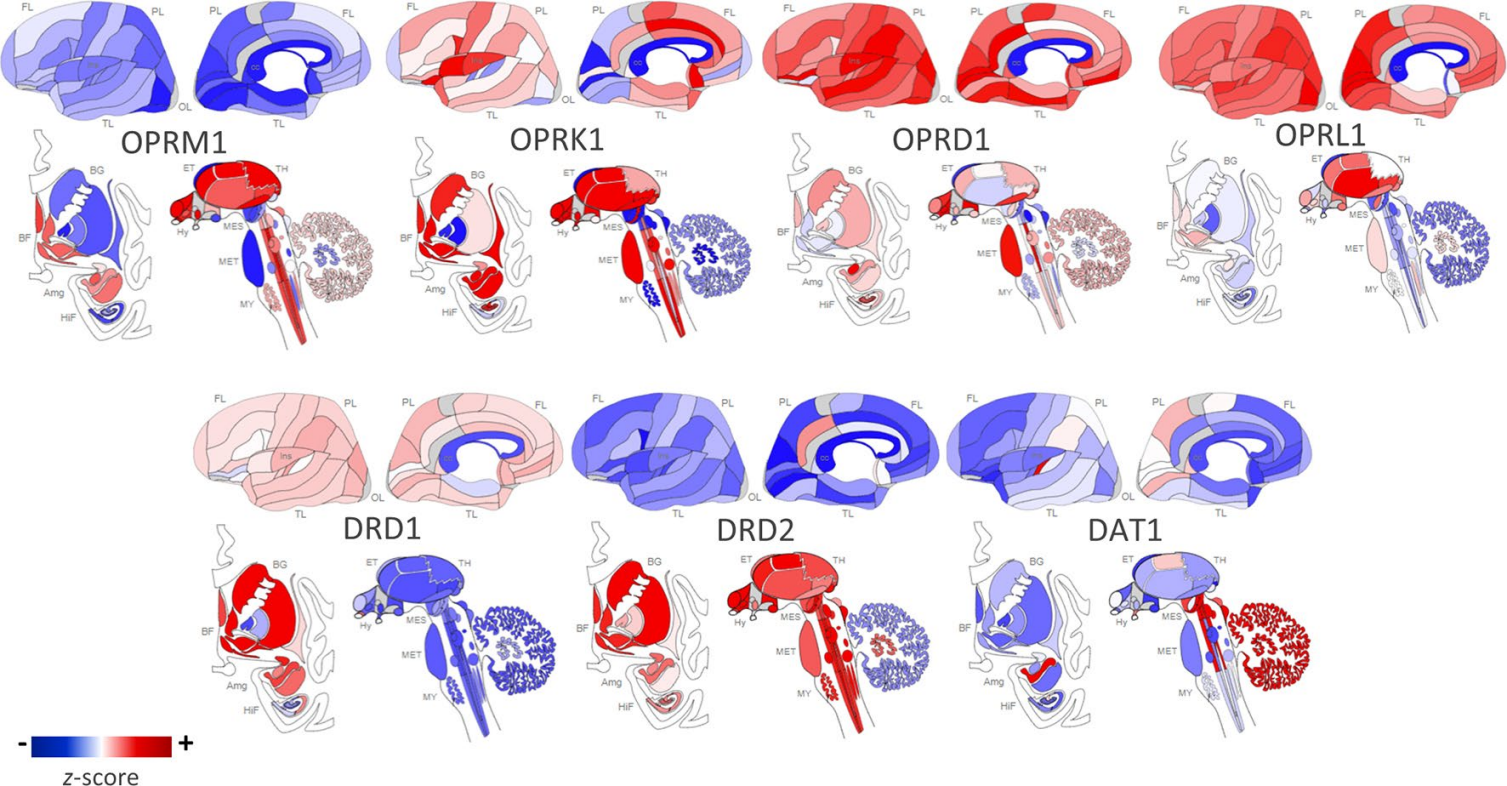
Regional distribution of receptor types in the human brain. Opioid and dopamine receptor gene expression in the human brain [Opioid Receptor Mu 1 (OPRM1), Opioid Receptor Kappa 1 (OPRK1), Opioid Receptor Delta 1 (OPRD1), Opioid Related Nociceptin Receptor 1 (OPRL1), Dopamine Receptor D1 (DRD1), Dopamine Receptor D2 (DRD2), Dopamine Active Transporter 1 (DAT1)]. Images constructed using Allen Human Brain Atlas. Data displayed are from one donor: H0351.2002, 39 years, M, Black or African American. The color bar displays expression values using *z*-score normalization. Color scale was altered to highlight regional differences in gene expression per receptor type; therefore, the absolute scale differs across each of the receptor subtypes. For quantitative results from all six postmortem donor brains, visit http://human.brain-map.org/static/brainexplorer.

The *OPRM1* gene codes for the MOP receptor, an inhibitory G-protein coupled receptor (GPCR) that binds endogenous opioid peptides such as β-endorphin and enkephalins as well as exogenous opioids such as morphine and heroin (67). MOP receptors are required to establish morphine place preference and physical dependence (68). MOP receptors are expressed throughout the brain’s reward pathways including the mesocorticolimbic network as illustrated in **Figure 1**; their proposed mechanism for positive reinforcement in OUD is through disinhibition of DA neurons that trigger drug reward upon DA release (69, 70). Originally it was thought that MOP receptor agonists hyperpolarize GABAergic interneurons of the ventral tegmental area (VTA), reducing GABA-mediated inhibitory input to DA neurons and thereby increasing DA signaling by disinhibition (69). However, most evidence now suggests that the rostromedial tegmental nucleus mediates opioid-induced disinhibition of DA neurons (71–73). There is preclinical evidence of DA-independent opioid-induced reward, but the mechanism is not well understood (74, 75).

The effects of prolonged opioid exposure on MOP receptors, whether in the context of chronic pain management or substance abuse, are not fully understood. Bolger et al. (76) demonstrated an upregulation in MOP receptor in rat brain after chronic heroin administration. However, several other studies have demonstrated that both morphine and buprenorphine administration downregulate MOP receptors in rat brain (76, 77) including striatum (78). Clinically, prolonged exposure to opioids results in tolerance and increased opioid dose requirements; several proposed mechanisms may explain this phenomenon, including phosphorylation and arrestin-driven uncoupling of the GPCR and receptor internalization and degradation (79–82). However, several studies cloned MOP receptors in human embryonic kidney cells and found that morphine does not promote MOP receptor endocytosis (80, 83–85), which results in protracted desensitization that could contribute to tolerance (86). Yet, several opioids including methadone, etorphine, and [D-Ala2, N-MePhe4, Gly-ol]-enkephalin (DAMGO) induced the expected receptor sequestration in cell line models (79, 80, 87, 88). A study in rats also showed MOP receptor internalization in the striatum and habenula after acute etorphine, but not morphine administration (80). These findings were replicated in the rat’s locus coeruleus where neurons showed MOP receptor internalization in response to DAMGO and methadone, but not morphine (89). Downregulation of MOP receptors is agent-specific as some opioids are more effective at activating the G-protein response than others (87). The concept of biased agonism explains differential activation patterns and intracellular signaling cascades based on ligand structure and GPCR conformations (90, 91). In the case of MOP receptors, ligands may preferentially activate G-protein coupling or β-arrestin recruitment (92). Schmid et al. (92) reported that fentanyl promotes bias toward β-arrestin recruitment, while morphine is relatively unbiased in mouse models and cell lines. Given that β-arrestin drives MOP receptor internalization and is associated with respiratory suppression and tolerance, these findings have clinical significance and may explain the differences in ligand-mediated MOP receptor internalization (92–95). Specifically, the increased lethality of fentanyl and structurally related synthetic opioids may not be due solely to greater potency, but also due to the preferential activation of an intracellular pathway that promotes respiratory depression (92, 96).

#### *OPRM1* Polymorphisms

Genetic variations of *OPRM1*, the gene encoding for MOP, have been studied in the context of vulnerabilities to SUDs, treatment response, and relapse. Whole genome sequencing has identified 3,324 *OPRM1* polymorphisms, the most commonly studied of which, rs1799971 (A118G), has a global minor allele frequency of 19% (97). Located on exon 1 of *OPRM1*, this SNP results in an asparagine replaced by an aspartate at position 40, which is in the amino-terminus of the receptor (98, 99). In preclinical studies, the G allele was associated with lower MOP receptor expression in transfected cell lines (100–103). In [^11^C]carfentanil PET scans, the G allele was also associated with lower global MOP receptor expression (104) and lower expression in anterior cingulate cortex (ACC), nucleus accumbens (NAc), and thalamus compared to the common genotype (105). One proposed mechanism suggests that the amino acid substitution removes an extracellular glycosylation site, potentially interfering with the protein’s folding or incorporation into the cell membrane (101). Other studies found that the G allele results in reduced levels of MOP receptor mRNA expression, although the underlying mechanism remains unknown (103). For example, a post-mortem study of heterozygotes for A118G found the wild-type A allele had twice the mRNA expression than the G variant in cortical and pons tissue samples (103). An *in vitro* study of G allele-transfected cells also showed reduced mRNA and lower receptor protein levels when compared to the wild-type allele (103). Oertel et al. (106) propose that rs1799971 creates a novel methylation site that suppresses transcription of *OPRM1*.

Interestingly, an initial *in vitro* study reported increased binding affinity of β-endorphin to the variant receptor (107); though subsequent *in vitro* studies were unable to replicate this finding (100, 108).

#### Genetic Association Studies: *OPRM1* and OUD

Several studies have investigated the effects of genetic variations in *OPRM1* on susceptibility to SUDs, including OUD. A systematic review and meta-analysis of 13 studies of the A118G polymorphism in OUD found significant associations of the G allele with CUD and OUD in Asian populations, but not in African American, Caucasian, or Hispanic populations (109). However, a behavioral study linked the G allele with increased addiction severity in Caucasian males with OUD (110). This could be attributable to the varying prevalence of the rs1799971 minor allele across ethnicities; for example, the G allele frequency is greater in Asian populations than in Caucasians (30–40% and 11–15%, respectively), and it is less than 5% in African American populations (107, 111, 112). Another study examined four low-frequency SNPs of *OPRM1* in a cohort of European Americans and African Americans; only one polymorphism, rs62638690, was associated with both cocaine and heroin addiction in European Americans; however, it did not withstand correction for multiple testing (113). This may suggest that while *OPRM1* polymorphisms alter vulnerability to OUD, the effects are race- and/or ethnicity-dependent. Finally, an intron 2 polymorphism, rs9479757, was not associated with OUD in a Chinese population, but OUD patients with the minor allele were found to consume higher levels of opioids (114). Further, Xu et al. (115) found the rs9479757 minor allele associated with addiction severity among Chinese OUD patients (115). These findings are outlined in **Table 1**.

**Table 1:** Polymorphisms associated with OUD in the opioid system and molecular imaging correlates.

Gene	Polymorphism	Location	Finding	Author	Year	n	Ethnicity	Imaging Correlates
***OPRM1***	**rs1799971**	Exon 1	Risk factor for OUD	Kumar et al. (116)	2012	330	Indian	-Lower baseline MOP receptor binding potential in NAc and amygdala of tobacco smokers (117–119) -Greater DA release in the right caudate and ventral pallidum in response to smoking (120)
				Kapur et al. (121)	2007	282	Indian	
				Deb et al. (122)	2010	169	Indian	
				Tan et al. (123)	2003	137	Indian	
				Nagaya et al. (124)	2012	160	Malaysian males	
				Szeto et al. (111)	2001	296	Chinese	
				Bart et al. (125)	2004	309	Caucasian	
				Drakenberg et al. (126)	2006	65	Caucasian	
			No significant association with OUD	Bond et al. (107)	1998	31	African American	
				Luo et al. (127)	2003	100	African American	
				Gelernter et al. (112)	1999	288	African American	
				Crowley et al. (128)	2003	195	African American	
				Zhang et al. (40)	2006	600	Caucasian	
				Bond et al. (107)	1998	52	Caucasian	
				Gelernter et al. (112)	1999	492	Caucasian	
				Franke et al. (129)	2001	652	Caucasian	
				Luo et al. (127)	2003	231	Caucasian	
				Crowley et al. (128)	2003	229	Caucasian	
				Levran et al. (130)	2008	596	Caucasian	
				Nikolov et al. (131)	2011	3,283	Caucasian	
				Bond et al. (107)	1998	67	Hispanic	
				Gelernter et al. (112)	1999	94	Hispanic	
				Li et al. (132)	2000	434	Chinese	
				Zhang et al. (133)	2007	332	Chinese	
				Shi et al. (114)	2002	145	Chinese	
				Tan et al. (123)	2003	208	Chinese	
				Tan et al. (123)	2003	156	Malay	
			No significant association with methadone dose	Crettol et al. (134)	2008	238	Caucasian	
			Prolonged abstinence without agonist therapy	Levran et al. (135)	2017	596	Caucasian	
	**rs62638690**	Exon 2	Protective against OUD	*Clarke et al. (113)	2013	1,377	European American	
	**rs510769**	Intron 1	Risk factor for OUD	*Levran et al. (130)	2008	596	Caucasian	
	**rs3778151**	Intron 1	Risk factor for OUD	*Levran et al. (130)	2008	596	Caucasian	
	**rs9479757**	Intron 2	Higher opioid consumption	Shi et al. (114)	2002	145	Chinese	
			Addiction severity	Xu et al. (115)	2014	332	Male Chinese	
***OPRD1***	**rs569356**	Promoter	Risk factor for OUD	*Zhang et al. (136)	2008	1,063	European American	
			No significant association with OUD	Nelson et al. (137)	2014	2,954	Australian	
	**rs4654327**	3’ UTR	Risk factor for OUD	Gao et al. (138)	2017	774	Chinese	
			No significant association with OUD	Nelson et al. (137)	2014	2,954	Australian	
	**rs1042114**	Exon 1	Risk factor for OUD	Nagaya et al. (139)	2018	1,002	Malay males	
				Zhang et al. (136)	2008	1,063	European American	
				Crist et al. (140)	2013	566	Caucasian males	
			No significant association with OUD	Nelson et al. (137)	2014	2,954	Australian	
	**rs2234918**	Exon 3	Risk factor for OUD	Huang et al. (141)	2018	1,331	Chinese	
				Mayer et al. (142)	1997	218	Caucasian	
			No significant association with OUD	Xu et al. (143)	2002	754	Chinese	
				Levran et al. (130)	2008	596	Caucasian	
				Zhang et al. (136)	2008	1,063	European American	
				Franke et al. (144)	1999	406	Caucasian	
				Crist et al. (140)	2013	2,502	Mixed	
			No significant association with methadone dose	Crettol et al. (134)	2008	455	Caucasian	
	**rs508448**	Intron 1	Earlier onset OUD	Gao et al. (138)	2017	774	Chinese	
			No significant association with OUD	Nelson et al. (137)	2014	2,954	Australian	
	**rs581111**	Intron 1	Risk factor for OUD	Crist et al. (140)	2013	1,006	African American	
			Higher relapse rates on buprenorphine	Clarke et al. (145)	2014	582	Caucasian females	
			No significant association with OUD	Nelson et al. (137)	2014	2,954	Australian	
	**rs678849**	Intron 1	Risk factor for OUD	Sharafshah et al. (146)	2017	404	Iranian	
			Abstinence-induced withdrawal severity	*Jones et al. (147)	2016	19	Mixed	
			Higher relapse rates on buprenorphine	Crist et al. (148)	2013	77	African American	
				Crist et al. (149)	2018	55	African American	
			Lower relapse rates on methadone	Crist et al. (148)	2013	77	African American	
			No significant association with relapse rates on methadone	Crist et al. (149)	2018	55	African American	
			No significant association with OUD	Nelson et al. (137)	2014	2,954	Australian	
				Zhang et al. (136)	2008	1,063	European American	
	**rs2236857**	Intron 1	Risk factor for OUD	Sharafshah et al. (146)	2017	404	Iranian	
				Nelson et al. (137)	2014	2,954	Australian	
				*Levran et al. (130)	2008	596	Caucasian	
			No significant association with OUD	Zhang et al. (136)	2008	1,063	European American	
			Protective against stress response in OUD	Huang et al. (141)	2018	1,331	Chinese	
	**rs2236857**^+^ **rs581111 haplotype**^†^	Intron 1	Risk factor for OUD	Nelson et al. (137)	2014	2,954	Australian	
	**rs2236855**	Intron 1	Risk factor for OUD	Sharafshah et al. (146)	2017	404	Iranian	
				Nelson et al. (137)	2014	2954	Australian	
			No significant association with OUD	Zhang et al. (136)	2008	1,063	European American	
				Crist et al. (140)	2013	566	Caucasian males	
	**rs760589**	Intron 1	Risk factor for OUD	Sharafshah et al. (146)	2017	404	Iranian	
				*Nelson et al. (137)	2014	2,954	Australian	
	**rs2236861**	Intron 1	Risk factor for OUD	Beer et al. (150)	2013	284	Western European	
				*Levran et al. (130)	2008	596	Caucasian	
				*Nelson et al. (137)	2014	2,954	Australian	
	**rs529520**	Intron 1	Higher methadone requirement	Luo et al. (151)	2017	257	Chinese	
			Higher relapse rates on buprenorphine	Clarke et al. (145)	2014	582	Caucasian females	
			Risk factor for OUD	*Nelson et al. (137)	2014	2,954	Australian	
			No significant association with OUD	Zhang et al. (136)	2008	1,063	European American	
	**rs10753331**	Intron 1	Abstinence-induced withdrawal severity	Jones et al. (147)	2016	19	Mixed	
			Risk factor for OUD	Crist et al. (140)	2013	566	Caucasian	
	**rs3766951**	Intron 1	Risk factor for OUD	Nelson et al. (137)	2014	2,954	Australian	
				*Levran et al. (130)	2008	596	Caucasian	
	**rs2298897**	Intron 1	Risk factor for OUD	Nelson et al. (137)	2014	2,954	Australian	
**OPRK1**	**rs1051660**	Exon 2	Risk factor for OUD	Yuferov et al. (152)	2004	291	Mixed	
				Gerra et al. (153)	2007	176	Caucasian Italian	
	**rs702764**	Exon 4	No significant association with OUD	Nagaya et al. (139)	2018	1,002	Malay males	
				Zhang et al. (136)	2008	1,063	European American	
	**rs997917**	Intron 2	Risk factor for OUD	Albonaim et al. (154)	2017	404	Iranian	
			No significant association with OUD	Zhang et al. (136)	2008	1,063	European American	
	**rs6985606**	Intron 2	Risk factor for OUD	Albonaim et al. (154)	2017	404	Iranian	
			No significant association with OUD	Zhang et al. (136)	2008	1,063	European American	
	**rs6473797**	Intron 2	Protective against OUD	*Levran et al. (130)	2008	596	Caucasian	
			Naloxone-precipitated withdrawal severity	Jones et al. (147)	2016	29	Mixed	
			No significant association with OUD	Albonaim et al. (154)	2017	404	Iranian	
***PDYN***	**rs35286281** **H allele**	Promoter	Risk factor for OUD	Yuanyuan et al. (155)	2018	1,107	Chinese	
				Wei et al. (156)	2011	604	Chinese	
			No significant association with OUD	Hashemi et al. (157)	2018	435	Iranian	
	**rs1997794**	Promoter	Risk factor for OUD	Clarke et al. (158)	2012	2,618	European American females	
				Clarke et al. (159)	2009	858	Chinese females	
			No significant association with OUD	Nagaya et al. (139)	2018	1,002	Malaysian males	
	**rs2281285**	Intron 2	No significant association with OUD	Hashemi et al. (157)	2018	435	Iranian	
	**rs910080**	3’ UTR	Risk factor for OUD	Nagaya et al. (139)	2018	1,002	Malaysian males	
				Clarke et al. (158)	2012	2,618	European American females	
				Wei et al. (156)	2011	604	Chinese	
				Hashemi et al. (157)	2018	435	Iranian	
			No significant association with OUD	Clarke et al. (158)	2012	2,618	European American males	
	**rs1022563**	3’ UTR	Risk factor for OUD	Clarke et al. (158)	2012	2,618	European American females	
				Clarke et al. (159)	2009	858	Chinese females	
				Wei et al. (156)	2011	604	Chinese	
			No significant association with OUD	Nagaya et al. (139)	2018	1,002	Malaysian males	
	**rs2235749**	3’ UTR	Risk factor for OUD	Wei et al. (156)	2011	604	Chinese	
			No significant association with OUD	Hashemi et al. (157)	2018	435	Iranian	
***OPRL1***	**rs6512305**	Intron 1	Risk factor for OUD	*Xuei et al. (160)	2008	1,923	European American	
	**rs6090043**	Intron 1	Risk factor for OUD	*Xuei et al. (160)	2008	1,923	European American	
			No significant association with OUD	Briant et al. (161)	2010	447	African American	
	**rs6090041**	Intron 1	Risk factor for OUD	Briant et al. (161)	2010	447	Caucasian	
			No significant association with OUD	Briant et al. (161)	2010	447	African American	
				Xuei et al. (160)	2008	1,923	European American	
	**rs6090043**^+^ **rs6090041 haplotype**^‡1^	Intron 1	Risk factor for OUD	Briant et al. (161)	2010	447	Mixed	
	**rs6090043**^+^ **rs6090041 haplotype**^‡2^	Intron 1	Risk factor for OUD	Briant et al. (161)	2010	447	Caucasian	
			No significant association with OUD	Briant et al. (161)	2010	447	African American	

SNP associations refer to the minor allele.

*Nominal significance.

† rs2236857 + rs581111 GA haplotype (coupled minor alleles).

‡^1^ rs6090043 + rs6090041 AT haplotype.

‡^2^ rs6090043 + rs6090041 GC haplotype.

Additionally, the A118G polymorphism may have relevance for OUD treatment. In a mouse model of A118G, the analgesic, anxiolytic, and hyperlocomotor effects of buprenorphine were attenuated in carriers of the minor G allele (162). In a study of opioid-dependent chronic pain patients, carriers of the minor G allele required higher morphine equivalent daily doses than AA homozygotes (163). This may be attributed to reduced MOP receptor functioning in carriers of the G allele that results in an increased opioid requirement for pain management (163, 164). However, a meta-analysis of the association between rs1799971 and methadone treatment response among OUD patients was inconclusive (165).

Several studies have examined associations between *OPRM1* polymorphisms and stress response, as MOP receptors help regulate stress levels *via* tonic inhibition of the hypothalamic–pituitary–adrenal (HPA) axis (166). Naloxone is an opioid receptor antagonist with highest affinity for MOP receptors, thus eliciting an HPA axis stress response upon binding (167). Several studies demonstrate that healthy heterozygotes of A118G have increased stress response to naloxone compared to non-G allele carriers (168–170). Given the role of stress dysregulation in vulnerability to SUDs, this provides a potential mechanism for this SNP as a risk factor for OUD (167).

The A118G SNP has also been associated with personality traits relevant to SUDs (171). Several studies assessed participants with the five-factor NEO, a personality inventory that scores in domains of “Openness to Experience, Conscientiousness, Extraversion, Agreeableness, and Neuroticism” (172). High Neuroticism, low Conscientiousness, and low Agreeableness scores are associated with SUDs (173–176). Specifically, higher scores on Neuroticism and lower scores on Conscientiousness, Agreeableness, and Extraversion have been associated with OUD (177, 178). Compared to A118 homozygotes, carriers of the G allele scored lower on the Conscientiousness factor (170), which is associated with task organization and execution, and reflects control over impulsivity (179). Moreover, Pecina et al. (105) found that G carriers had higher Neuroticism scores than non-carriers, which negatively correlated with baseline MOP receptor availability in the anterior insula and subgenual ACC as assessed with [^11^C]carfentanil PET. However, Hernandez-Avila et al. (180) found no association between A118G and NEO personality dimensions in healthy and substance-dependent volunteers; thus, the role of this polymorphism in moderating personality is uncertain. Love et al. (181) used [^11^C]carfentanil PET in a study of healthy volunteers and assessed participants with the Revised NEO Personality Inventory, which includes domains “Impulsiveness” and “Deliberation,” that have been associated with negative risk-taking, including drug use (182, 183). Participants with high Impulsivity and low Deliberation scores showed higher baseline MOP receptor availability in several brain regions including the ACC and amygdala (181). Further, in response to a pain stress challenge, subjects with high Impulsivity/low Deliberation scores demonstrated a larger reduction in MOP receptor availability from baseline compared to low Impulsivity/high Deliberation scores in regions including the orbitofrontal cortex and amygdala (181). This suggests a possible mechanism for the role of personality traits in shaping vulnerabilities to SUDs.

#### Molecular Imaging: MOP Receptor and OUD

Several studies have used PET imaging to investigate MOP receptor availability in OUD patients receiving MOUD. The radioligand [^11^C]carfentanil is widely used in PET studies as it is a highly potent MOP-selective receptor agonist (184). [^18^F]cyclofoxy is less frequently used as it is both a MOP receptor and KOP receptor agonist, with some preliminary evidence of MOP receptor preference (185–188).

A number of studies have examined the effects of buprenorphine, a high-affinity MOP receptor partial agonist and KOP and delta opioid (DOP) receptor antagonist (189–191) in the treatment of OUD. Using [^11^C]carfentanil PET imaging, Greenwald et al. (192) investigated the duration of binding of buprenorphine at MOP receptor and the corresponding effects on withdrawal in 10 OUD patients. They found that 50–60% MOP receptor occupancy by buprenorphine was required for withdrawal suppression (192). At 28 h after buprenorphine, 46% of whole-brain MOP receptors were occupied, indicating inadequate withdrawal suppression (192). This may reflect the half-life of oral buprenorphine, which ranges from 28 to 37 h (193). Plasma concentrations of buprenorphine were time-dependent and correlated with levels of MOP receptor occupancy in brain (192, 194). Considering the minor allele of rs1799971 may lower MOP receptor expression, it stands to reason that this SNP may influence the dose of buprenorphine required to achieve adequate withdrawal suppression.

In two studies, heroin-dependent patients maintained on varying doses of buprenorphine underwent several [^11^C]carfentanil PET scans (194, 195). Buprenorphine was shown to reduce MOP receptor availability in a dose-dependent manner, and decreased MOP receptor availability correlated with decreased heroin craving and withdrawal symptoms (194, 195). After detoxification from buprenorphine, OUD participants demonstrated higher regional binding potential of MOP receptor particularly in the inferior frontal and anterior cingulate cortex compared to healthy controls (195). Yet, an animal study found buprenorphine maintenance down-regulates MOP receptor in rat brains (77). The higher MOP receptor binding potential among OUD participants found by Zubieta et al. (195) could reflect opioid or buprenorphine induced downregulation of enkephalins and β-endorphins in brain with a consequent reduced competition for [^11^C]carfentanil binding to MOP.

Another study used [^18^F]cyclofoxy PET scans in 14 methadone-maintained patients and 14 healthy controls (185). The methadone-maintained patients demonstrated 19–32% lower cyclofoxy binding than the controls in thalamus, caudate, anterior cingulate cortex, middle temporal cortex, and the middle frontal cortex (185). The lower [^18^F]cyclofoxy binding in the brain of OUD participants correlated with plasma methadone levels, likely reflecting the steady-state methadone occupancy of MOP receptors (185). These findings contrast with those obtained in OUD patients treated with buprenorphine who showed much greater levels of MOP occupancy consistent with the partial agonist effects of buprenorphine as compared to the full agonist effects of methadone (192). This discrepancy could also reflect less receptor internalization associated with a partial agonist and, therefore, greater levels of receptor occupancy by the radioligand.

PET studies have also investigated the effects of A118G on MOP receptor availability in individuals with SUDs. For example, the G allele has been associated with lower baseline MOP receptor binding potential in NAc and amygdala of smokers (146–148). Thus, A118G may shape predispositions to substance abuse by affecting MOP receptor availability, which could contribute to aberrant dopaminergic signaling. A [^11^C]raclopride PET study of tobacco smokers found that the G allele associated with greater DA release in the right caudate and ventral pallidum in response to smoking compared to the A allele (120). This is further evidence of the association between A118G and drug reward, which may increase vulnerability to SUDs (120). Longitudinal studies are needed to clarify the link between opioid receptor availability and SUDs.

### OPRK1

#### *OPRK1* Background

*OPRK1* codes for the KOP receptor, an inhibitory GPCR that is implicated in the brain’s stress or anti-reward system (196). KOP receptors are the most abundant opioid receptors in the human brain and are highly expressed in key brain regions of the stress axis such as the prefrontal cortex and amygdala (197) as well as in reward-related regions including the VTA, NAc core, dorsal striatum, and substantia nigra as seen in **Figure 1** (187, 198–201). KOP receptors are coupled with calcium channels and are localized in presynaptic terminals of dopaminergic cells; activation of KOP receptors inhibits adenylyl cyclase and calcium currents, thereby inhibiting DA release (199, 202–204). Prodynorphin (*PDYN*) codes for the precursor to the dynorphin peptide, which is the endogenous ligand to the KOP receptor. Using a phospho-selective antibody against KOP receptors, Land et al. (16) demonstrated that both stress paradigms and CRF injections elicit dynorphin-dependent KOP receptor activation in the basolateral amygdala, NAc, and hippocampus of mice. This indicates the key role KOP receptor signaling plays in stress and dysphoria. In general, KOP receptor agonists have anxiogenic properties in humans (205, 206) while KOR antagonists demonstrate anxiolytic properties in animal models (207, 208). However, there is evidence of dose-dependent effects; in a mouse study, KOP receptor agonist, U50,488H, was anxiolytic at high doses but anxiogenic in low doses (209). KOP receptor signaling may also influence stress responses associated with relapse; for example, heroin-dependent rats treated with KOP receptor antagonists show reduced anxiety- and stress-induced reinstatement of drug-seeking behavior (210, 211).

KOP receptor signaling is also involved in an array of physiological functions such as mood modulation, pain perception, learning and memory, and behavioral response to drugs of abuse (212, 213). Within the NAc, dynorphin signaling inhibits DA release, which leads to aversive effects on mood (214). In individuals with SUDs, KOP receptor-mediated dynorphin signaling drives negative affective states during drug withdrawal (215). One [^11^C]raclopride PET study showed blunted DA release with a methylphenidate challenge in recently detoxified OUD patients compared to healthy controls (7). This hypodopaminergic response may be explained by dynorphin-mediated withdrawal. This is consistent with a rodent study that found chronic exposure and subsequent withdrawal from morphine led to prolonged (15 day) decreases in spontaneous dopaminergic neuron activity (216). This hypodopaminergic state may underlie dysphoria that drives compulsive drug-seeking (216).

Interestingly, post-mortem brain samples of heroin abusers showed lower levels of PDYN mRNA expression in the amygdalar nucleus of the periamygdaloid cortex compared to controls (217). Further, a post-mortem study reported elevated dynorphin levels in heroin abusers with reduced striatal PDYN mRNA expression, suggesting upregulation of PDYN mRNA translation despite reduced PDYN mRNA levels (126). These results corroborate findings of reduced PDYN mRNA expression and elevated expression of the brain stress marker, CRF, in the periamygdaloid cortex of heroin-dependent rats that were euthanized following 24 h of abstinence (217). Increased CRF may reflect the dynorphin-mediated withdrawal response in the heroin-dependent rats despite seemingly reduced *PDYN* expression (217).

Preclinical studies have found that KOP receptor agonists, including salvinorin A, cause KOP receptor internalization *in vitro* (218, 219). A [11C]GR103545 PET study in rodents found that a dose of 0.60 mg/kg of salvinorin A resulted in a prolonged decrease in [11C]GR103545 binding that persisted even after salvinorin A had cleared from the brain, consistent with KOP receptor internalization (220). This study provides insight into the neurochemical adaptations to KOP receptor agonist exposure, which may contribute to opioid tolerance (18).

#### *OPRK1* Polymorphisms

A few *OPRK1* polymorphisms have been described in the context of SUDs, although the majority of them are silent and have no effect on gene expression (221). One example is rs1051660 (G36T), a synonymous SNP in exon 2 (153). These polymorphisms may affect KOP receptor signaling indirectly by altering mRNA stability or translation (222).

*PDYN* polymorphisms are associated with aberrant dynorphin expression and signaling (223) that may contribute to dysphoria and relapse during opioid withdrawal (155). Intronic variants may alter gene expression *via* splicing mechanisms or may be in linkage disequilibrium with neighboring variants that have more direct downstream effects (146, 224). Mutations within the 3’ tail of mRNA transcripts could alter important sequences like the polyadenylate tail and may disrupt transcription termination (225), translation, and stability of mRNA (226–228). For example, rs910080, a polymorphism in the 3’ untranslated region of *PDYN*, is in high linkage disequilibrium with two other 3’ untranslated region SNPs, rs910079 and rs2235749; in a post-mortem analysis, this haplotype block was associated with levels of PDYN expression in the striatum (229). Other polymorphisms may alter gene expression directly. The 68-base pair variable number tandem repeat (VNTR) polymorphism, rs35286281, ranges from two to five repeats in the promoter region of *PDYN*, with each repeat containing one binding site for a transcription factor (230, 231). Thus, high dynorphin expression alleles (H alleles) contain three or more repeats and are associated with higher *PDYN* transcription and translation compared to low dynorphin expression alleles (L alleles) with one or two repeats (230).

#### Genetic Association Studies: *OPRK1*, *PDYN*, and OUD

There has been little consensus regarding the role of *OPRK1* polymorphisms in OUD. The minor alleles of two intronic polymorphisms, rs997917 and rs6985606, were reported as risk factors for OUD in an Iranian population (154) but were not associated with OUD in a European American population (136). These conflicting findings are likely explained by ethnicity-dependent effects. Interestingly, the rs6473797 minor allele was found to be protective against OUD in a Caucasian population (130), but not in an Iranian population (154). However, rs6473797 did associate with withdrawal severity among OUD patients who underwent naloxone-precipitated withdrawal in an American population of mixed ethnicities (147). Additionally, Wang et al. (232) found that two *OPRK1* haplotype blocks associated with withdrawal symptoms such as joint aches, gooseflesh skin, and yawning in Taiwanese methadone-maintained OUD patients. Lastly, rs1051660 was initially linked to OUD (152), and this finding was replicated by Gerra et al. (153) in a Caucasian Italian population.

Given the critical role of dynorphin signaling in the negative emotion states of SUDs, several studies have examined *PDYN* polymorphisms in the context of OUD. One polymorphism, rs910080, has been associated with OUD across a wide range of ethnicities (139, 156–158). Additionally, there is evidence of sex effects on the association between another two *PDYN* polymorphisms and OUD. That is, both rs1997794 and rs1022563 were found to associate with OUD among European American females, but not males (158). In a prior study of Chinese females, Clarke et al. (159) found the rs1997794 minor allele associated with OUD. Further, these two polymorphisms were not associated with OUD in a study of Malaysian males (139). Together, these findings suggest sex- and ethnicity-specific effects of the *PDYN* genotype on susceptibility to OUD.

Two studies found that the H allele of the *PDYN* VNTR polymorphism was a risk factor for OUD in Chinese populations (155, 156). It was also associated with greater instances of withdrawal and subsequent relapse among heroin-dependent Chinese patients on methadone therapy (155). However, Hashemi et al. (157) did not find an association between the *PDYN* genotype and OUD in an Iranian population. While evidence exists that the H allele upregulates *PDYN* expression (230), further research is required to understand its functional consequences as it relates to OUD.

Despite preclinical and clinical evidence of KOP receptor signaling modulating anxiety and stress response (16, 205, 206, 210, 211, 233, 234), few studies have investigated the effects of *OPRK1* polymorphisms on personality or behavior. One study using the five-factor NEO found the minor allele at rs963549, in exon 3 of *OPRK1*, was associated with higher Neuroticism scores among participants with SUDs but not among healthy controls (235). While this SNP was found to not be a risk factor for SUDs in an Indian population (116), its effects may be ethnicity-dependent or potentially mediated by opioid use. Future studies on the functional effects of *OPRK1* polymorphisms and their associated changes in neurochemistry and behavior would clarify the link between KOP receptor signaling and OUD.

One study examining the effects of the *PDYN* VNTR polymorphism on behavior found that the L allele is associated with disinhibited behavior as assessed with the Zuckerman Sensation Seeking Scale (236). Given that higher scores on this scale correlate with a preference toward risky behavior, this finding suggests L allele carriers are at increased risk for SUDs, contradicting findings from genetic association studies described above (155, 156) but perhaps corroborating post-mortem findings of reduced *PDYN* expression in individuals with OUD (217).

#### Molecular Imaging: KOP Receptor and OUD

At this point, no studies have used PET to examine *OPRK1* polymorphisms among patients with OUD. Only recently have radiotracers been developed to target KOP receptors, including the agonist tracers [^11^C]GR103545 and [^11^C]EKAP and the antagonist tracer [^11^C]LY2795050. These radiotracers have been evaluated in primates (237–240) and humans (241–244).

In a [^11^C]LY2795050 PET study, patients with AUD showed lower KOP receptor availability in the amygdala and pallidum compared to healthy controls (245). It is possible that the reduction in KOP receptor availability helps restore dopaminergic signaling and thus alleviates the aversive effects of drinking. However, reduced [^11^C]LY2795050 specific binding to KOP receptors in AUD could also reflect increased competition for radiotracer binding from upregulation of dynorphin. Another [^11^C]LY2795050 PET study found that healthy male subjects had greater KOP receptor availability in several brain regions including ACC, frontal cortex, insula, and ventral pallidum compared to females (246). According to the “simple occupation theory,” the robustness of a drug response is directly proportional to the number of receptors occupied by the drug (247). This is consistent with the finding by Vijay et al. (246) that greater KOP receptor availability may mediate stronger responses to KOP receptor antagonists such as naltrexone treatment. Among patients with co-occurring cocaine and alcohol dependence, one study showed that naltrexone treatment reduced cocaine and alcohol use in men, but increased substance use in women (248). While sex differences in KOP receptor availability were not examined by Pettinati et al. (248), the authors suggest that receptor bioavailability and naltrexone treatment response may be sex-dependent. A potential non-neurochemical basis for the poorer treatment response in women compared to men is that women report higher rates of naltrexone-induced nausea, which results in lower medication compliance (249). However, it is important to note that other clinical studies found no sex differences of naltrexone treatment response in AUD (250, 251). Overall, these findings suggest that KOP receptor availability is associated with alcohol use and could potentially mediate the efficacy of KOP-targeted pharmacotherapies for AUD (245). Given the high comorbidity between AUD and OUD (252–254), these findings might have implications for opioid-antagonist treatment response in OUD.

### OPRD1

#### *OPRD1* Background

*OPRD1* codes for DOP receptors, which are also involved in the negative affect and withdrawal stage of addiction, albeit with inverse effects than KOP receptors. Specifically, greater DOP receptor signaling leads to improvements in negative emotional states (255). DOP receptor agonists have demonstrated antidepressant and anxiolytic effects in rodent models (256, 257). DOP receptors are highly expressed in cortical and limbic areas such as the hippocampus and amygdala, as well as basal ganglia and hypothalamus (258–260). DOP receptors are located on presynaptic terminals of GABAergic interneurons and have region-specific effects on cAMP production (261). While striatal DOP receptor activation is inhibitory and results in increased extracellular dopamine (262), DOP receptors located in the olfactory bulb, medial prefrontal cortex, and primary cultures of hippocampal neurons stimulate cAMP production thereby inhibiting dopamine release (263–265).

Studies suggest DOP receptors modulate the rewarding effects of drugs of abuse. Le Merrer et al. (197) report DOP receptor knockout has no effect on morphine self-administration but does impair place conditioning in mice. In another rodent study, DOP receptor knockout resulted in reduced morphine reward and tolerance (266). Further, DOP receptor antagonists block sensitization to conditioned rewarding effects of opioids (267), whereas agonists enhance conditioned place preference to morphine (268). In a mouse model of OUD, DOP receptor knockout was associated with increased anhedonia and dysphoria during heroin abstinence compared to the wild-type genotype (269). Thus, *OPRD1* polymorphisms that alter DOP receptor signaling may influence opioid withdrawal-associated stress response and relapse.

#### *OPRD1* Polymorphisms

Several polymorphisms of *OPRD1* have been studied in the context of SUDs. One, rs1042114 (G80T), results in an amino acid substitution from cysteine to phenylalanine in the N-terminus of the DOP receptor, and is proposed to disrupt DOP receptor maturation, leading to increased internalization of the receptor compared to wild type (270). The coding-region variant rs2234918 (T921C) is a synonymous polymorphism, that is, it does not cause a change in the coding amino acid, and has conflicting evidence for a role in OUD. Finally, rs569356, located in the promoter region, has been implicated in altered OPRD1 expression; Zhang et al. (271) found the G allele increased *OPRD1* transcription in transfected cell lines. Few other *OPRD1* polymorphisms have been described in terms of their functional effects; however, several have been assessed in genetic association studies.

#### Genetic Association Studies: *OPRD1* and OUD

Two polymorphisms in the coding region of *OPRD1* have been associated with OUD. The rs1042114 polymorphism has been found to be a risk factor for OUD in Malaysian males (139) and in Caucasian populations (136, 140). However, Nelson et al. (137) did not replicate these findings in Australian OUD patients. Rs2234918, a synonymous *OPRD1* polymorphism, has also been studied in OUD with conflicting findings. The minor C allele of this polymorphism was initially reported as a risk factor for OUD in a German (142) and Chinese population (272). However, several studies have failed to replicate this association (130, 136, 140, 143) including a study that examined a German population but used a family-based association approach to control for population stratification (144). Thus, it is uncertain what role, if any, these *OPRD1* polymorphisms play in increasing vulnerability to OUD.

Several polymorphisms in intron 1 of *OPRD1* have been studied in OUD, although their functional effects remain largely unknown. Two studies found an association between rs2236861 and OUD among Caucasian patients (137, 150). Levran et al. (130) also found that the rs2236861 minor allele increases the risk of heroin dependence; however, the association did not survive multiple testing, perhaps due to a small sample size. Another intron 1 polymorphism, rs2236857, was associated with OUD in Iranian- and European-descent populations (130, 137, 146). However, Zhang et al. (136) were unable to replicate this association in a study of European Americans. Interestingly, among Chinese OUD patients, carriers of the rs2236857 minor allele were found to have higher subjective stress responses than non-carriers as assessed with the Life Event Questionnaire (272). This suggests that *OPRD1* polymorphisms may disrupt stress responses that increase addiction vulnerabilities. The minor allele of rs581111, located in intron 1, has also been reported as a risk factor for OUD among Australians (137) and African Americans but not European Americans (140). Additionally, the minor allele of rs581111 has been associated with poor response to buprenorphine treatment among Caucasian females, but not males, suggesting ethnicity- and sex-dependent influences on genetic associations (145). Lastly, the minor allele of an *OPRD1* intron 1 polymorphism, rs3766951, was reported as a risk factor for OUD in Caucasian populations (130, 137).

In addition, several studies have investigated the effects of *OPRD1* polymorphisms on treatment outcomes in OUD. For example, the major allele of rs678849 has been associated with higher relapse rates among African American OUD patients undergoing buprenorphine treatment, as indicated by positive opioid urine tests (148, 149). Interestingly, the major allele was initially associated with lower relapse rates among African American OUD patients on methadone treatment (148), but this association was not replicated (149). Jones et al. (147) reported an association between rs678849 and abstinence-induced opioid withdrawal severity; however, it did not withstand a multivariate analysis. While the mechanism of action is unknown, these findings suggest that rs678849 may affect OUD treatment outcomes by potentially mediating withdrawal symptoms.

Several other *OPRD1* polymorphisms have been studied in association with OUD with conflicting results as seen in **Table 1**.

*OPRD1* polymorphisms have also been associated with behaviors related to the negative affect and withdrawal stage of OUD. In one study of Pakistani OUD patients, the minor G allele of rs569356 was strongly associated with increased serum cortisol levels, a marker of stress response (273). Given the preclinical evidence that this minor allele may increase *OPRD1* transcription (271), the minor G allele may affect DOP receptor expression and stress responses that could contribute to OUD. While Zhang et al. (136) found a nominally significant association between rs569356 and OUD in a European American population, no significant association was found in Australian and Pakistani populations (137, 273).

#### Molecular Imaging: DOP Receptor and OUD

No PET studies have examined neurochemical differences between carriers of *OPRD1* polymorphisms in OUD. The only DOP-selective radiotracer that has been developed for PET imaging in humans is N1’-([^11^C]methyl)naltrindole ([^11^C]MeNTI) (274).

PET studies investigating DOP receptor availability in healthy controls and AUD patients may provide insight into the functional effects of *OPRD1* polymorphisms in OUD. One [^11^C]MeNTl PET study found that patients with AUD had slightly greater DOP receptor availability compared to healthy controls in the cingulate, amygdala, insula, ventral striatum, putamen, caudate nucleus, globus pallidus, and thalamus; however, group differences did not reach statistical significance (275). Within the AUD group, DOP receptor availability in the caudate showed a positive association with recent alcohol drinking (275). However, Weerts et al. (275) did not report associations between DOP receptor availability and other behavioral measures of alcohol dependence or withdrawal. Another PET study in abstinent AUD patients demonstrated that while naltrexone completely blocked MOP receptor radioligand binding, it only partially blocked [^11^C]MeNTl binding and there was high interindividual variability in DOP receptor blockade (276). These findings could underlie interindividual differences in responses to naltrexone treatment in AUD that could translate to naltrexone treatment responses in OUD.

Additionally, one [^11^C]MeNTl PET study found a negative correlation between mesolimbic DOP receptor availability and total cortisol output over a 4-h period following naloxone in healthy controls, but not in recently abstinent AUD patients (277). Given that endogenous DOP receptor signaling improves negative emotional states (278), the dissociation of DOP receptor availability from naloxone-induced cortisol response in AUD may suggest that chronic alcohol abuse disrupts DOP-mediated stress signaling during alcohol withdrawal. Whether this is the case for OUD remains to be determined. Notably however, Lutz et al. (269) reported that DOP receptor signaling ameliorates opioid withdrawal in rodents, so together, these findings may suggest a shared mechanism for negative emotional states in opioid and alcohol withdrawal.

### OPRL1

#### *OPRL1* Background

The nociceptin opioid peptide (NOP) receptor is an inhibitory GPCR encoded by the Opioid Receptor-Like 1 gene (*OPRL1*) that has MOP, KOP, and DOP receptor structure homology and similar signaling cascades (279). However, the NOP receptor is pharmacologically distinct from classical opioid receptors. The NOP receptor is activated by nociceptin, and its effects are not blocked by the universal opioid antagonist naloxone (280, 281). NOP receptors are distributed throughout the amygdala, hippocampus, thalamus, and cortical processing areas (282) and have roles in both analgesia and hyperalgesia [reviewed in (283) and (284)]. NOP receptor signaling is also involved in processes including stress, anxiety, depression, cognition, and addiction (285–289).

Given the distribution of NOP receptors along the limbic region (290), it follows that NOP signaling is tied to stress signaling. For example, central injections of nociceptin in rats result in increased plasma stress hormone levels, reflecting activation of the HPA axis (291). However, there is also evidence that NOP receptors in extrahypothalamic brain regions exert anti-stress effects. For example, nociceptin injections in the central nucleus of the amygdala reduce anxiety behaviors in rodents exposed to restraint stress (292). Further, body restraint stress upregulates NOP receptor mRNA in the central nucleus of the amygdala and basolateral amygdala (292). In an electrophysiological study, nociceptin blocked CRF-induced GABAergic transmission in slices from the central nucleus of the amygdala; these effects were more pronounced in neurons from ethanol-dependent rodents (293). Additionally, nociceptin injections in the bed nucleus of the stria terminalis block CRF-induced anxiety behaviors in rodents (294, 295). Thus, the role of NOP receptors in stress is likely complex and may be relevant in OUD, particularly due to the high co-occurrence of anxiety and SUDs [reviewed in (296)].

NOP receptor signaling also seems to have an anti-reward effect. In microdialysis studies, nociceptin administration was found to decrease extracellular DA levels in the NAc of anesthetized mice (297) and to decrease morphine-induced DA release in the NAc of rats (298). Further, in several rodent studies, NOP receptor agonists reduced conditioned place preference to alcohol, amphetamines, cocaine, and morphine, suggesting NOP receptor signaling may reduce the rewarding effects of these substances (299–304). However, Walker et al. (305) found nociceptin administration failed to reduce heroin self-administration in rodents. There is also preliminary evidence that the NOP receptor antagonist, LY2940094, could be efficacious in treating AUD in rodents and humans, perhaps by blocking stress-induced relapse (306, 307). While an initial post-mortem analysis demonstrated individuals with AUD had lower *OPRL1* expression in the central amygdala compared to controls (308), no difference in *OPRL1* expression was detected in another post-mortem study in individuals with SUDs including AUD (309). Thus, the NOP receptor is likely implicated in substance abuse and poses a potential therapeutic target, but further research is required to clarify its roles in reward and stress-related behaviors.

#### *OPRL1* Polymorphisms

The functional effects of several *OPRL1* polymorphisms have been studied. For example, two adjacent SNPs in intron 1, rs6512305 and rs6090043, are in high linkage disequilibrium and there is evidence that variants in rs6090043 may alter transcription factor binding sites, which could affect *OPRL1* gene expression (161). Further, the minor G allele at rs6090041, another intron 1 variant, and the minor C allele at rs6090043 provide additional transcription factor binding sites that could result in increased *OPRL1* transcription and NOP receptor availability (161). Given that NOP receptor signaling has been implicated in decreasing drug reward, there may be a role of *OPRL1* polymorphisms in susceptibility to SUDs.

#### Genetic Association Studies: *OPRL1* and OUD

Xuei et al. (160) assessed correlations between SUDs and polymorphisms in *OPRL1* as well as in the prepronociceptin gene (*PNOC*), which encodes the NOP receptor precursor, in a European American population; rs6512305 and rs6090043 were nominally associated with opioid dependence; however, no SNPs proved significant (160). Briant et al. (161) found that minor alleles at rs6090043 and rs6090041 were risk factors for OUD among Caucasians but not African Americans. One haplotype (AT) of these variants was found to be a risk factor in both Caucasians and African Americans, while another haplotype (GC) was a risk factor in Caucasians only (161). While there is preliminary evidence that *OPRL1* may influence vulnerability to OUD, further analysis is required to determine the potential ethnicity-dependent effects.

#### Molecular Imaging: NOP Receptor and OUD

NOP receptor antagonist PET radioligands have been developed; [^11^C]NOP-1A has been tested in humans (290, 310, 311) and [^18^F]MK-0911 has been tested in rhesus monkeys (312). To date, no molecular imaging of NOP has been done in participants with OUD; however, studies of other SUDs may provide insight. Using [^11^C]NOP-1A, Narendran et al. (313) found no difference in NOP receptor availability between healthy controls and recently abstinent AUD subjects, nor did NOP receptor availability correlate with clinical measures of addiction severity. This conflicts with preclinical evidence that NOP receptor signaling is involved with AUD (289, 299, 300, 308). However, the subjects with AUD in this study were abstinent for 16 to 54 days before the PET scan, and there is preclinical evidence that prolonged abstinence may recover NOP receptor levels in rats (313, 314). In another PET study, recently abstinent CUD participants demonstrated a significant increase in [^11^C]NOP-1A distribution volume notably in the midbrain, ventral striatum, and cerebellum compared to healthy controls (315). This increased NOP receptor availability may reflect a compensatory response to increased CRF transmission or decreased endogenous nociceptin associated with CUD (315). Further studies are required to evaluate NOP in OUD, for while studies in CUD have shown upregulation in brain, studies in AUD showed no differences (313), which suggests that there might be differences between SUDs. Also, research is needed to clarify changes during the different stages of the addiction cycle and to assess if there is recovery of NOP receptor availability with treatment.

## The Dopamine System

### DRD2

#### *DRD2* Background

The gene *DRD2* codes for D2R, an inhibitory GPCR distributed throughout the brain. Expression of D2R is concentrated in the basal ganglia nuclei, including the caudate, putamen, NAc, substantia nigra, and VTA, as shown in **Figure 1** (316). As such, D2R signaling plays an important role in cognition, reward, motivation, and drug addiction, including OUD (317, 318). MOP receptors are expressed on DA neurons in the reward pathway; thus, with opioid use, MOP receptor binding leads to a release of DA, which then binds striatal D2Rs, leading to a decrease in intracellular cAMP production (69, 319). This D2R signaling inhibits the indirect ventral striatal pathway, which is connected to punishment (320).

Ankyrin Repeat and Kinase Domain Containing 1 (*ANKK1*) is a gene directly downstream of *DRD2* on chromosome 11 that expresses a serine/threonine kinase (321). The protein product of *ANKK1* upregulates the expression of the transcription factor NF-κB (322). Increased NF-κB expression results in increased *DRD2* transcription (323).

Several studies have shown that OUD is associated with a disruption of the mesolimbic dopaminergic pathway, which underlies the behavioral response to opioids (4). Koob and Volkow (4) suggest that D2Rs contribute to drug seeking behaviors, but not drug reward directly (324, 325). A conditioned place preference study of *DRD2*-null mice demonstrated that D2Rs are in part responsible for the reinforcing nature of morphine (326).

Lower D2R levels observed in SUDs may reflect a homeostatic downregulation of D2R after excessive drug use (29), and some evidence exists that D2R levels increase after pronounced abstinence (327). Alternatively, lower D2R availability may be an inherent risk factor for drug abuse, even before the initiation of drug taking (328, 329).

#### *DRD2* Polymorphisms

A wide range of *DRD2/ANKK1* polymorphisms have been studied in the context of SUDs. One of the most well studied of these SNPs is *Taq*IA, located on exon 8 of *ANKK1*, adjacent to *DRD2* (321). Many studies have supported the role of *Taq*IA in addictive behaviors including various SUDs, obesity, and pathological gambling (330–333). Thus, the *Taq*IA1 variant, which alters ANKK1 substrate binding specificity, could lead to decreased D2R expression downstream (321). Indeed, [^11^C]raclopride and [^11^C]NMB PET studies have shown that minor alleles of *ANKK1* *Taq*IA and *Taq*IB, a linked *DRD2* SNP, are associated with low D2R availability in healthy controls (334–336). However, *Taq*IA is in linkage disequilibrium with several functional *DRD2* polymorphisms (337); thus, it is unclear if reduced D2R expression is associated with *Taq*IA directly.

Lesser studied *DRD2* variants may also contribute to OUD *via* a diminution of D2R expression (338). SNPs in the 5’ untranslated region of *DRD2*, including rs1799732, an insertion/deletion (*Ins/Del*) variant at position -141, have been shown to cause decreased promoter strength in an *in vitro* -141C *Del* luciferase construct (339). While one [^11^C]FLB-457 PET study found no association between rs1799732 and extrastriatal D2R in healthy volunteers (340), one [^11^C]raclopride PET study demonstrated higher striatal D2R availability in those with the combined minor variants of rs1799732, *Ins/Del* and *Del/Del*, compared to *Ins/Ins* (334). Until more studies are performed, the role of rs1799732 in D2R expression cannot be concluded.

Other *DRD2* polymorphisms produce splicing errors of the *DRD2* gene, resulting in altered D2R expression (341). For example, the minor allele of rs1076560, located in intron 6, is associated with a decreased ratio of short form D2 receptors (D2S) to long form receptors (D2L) (342). Preclinical studies have demonstrated that D2L knock-out mice have a loss of morphine preference in a conditioned place preference paradigm (343). Thus, this altered D2S/D2L ratio could help elucidate the mechanism of this SNP-OUD relationship. [^123^I]IBZM SPECT imaging revealed that in healthy volunteers, minor T allele carriers of this SNP showed lower levels of striatal D2R availability compared to G/G (344). However, another [^123^I]IBZM SPECT study in healthy volunteers did not replicate this finding (345). These findings may implicate *DRD2/ANKK1* polymorphisms in the lower D2R levels observed in individuals with OUD (6).

#### Genetic Association Studies: *DRD2* and OUD

Several polymorphisms in *DRD2/ANKK1* have been suggested to predispose OUD, as outlined in **Table 2**. Indeed, a recent meta-analysis across 11 studies, with a total sample of 4,529 OUD patients and 4,168 healthy controls, found that the *Taq*IA1 allele is a risk factor for (OUD) (354). Further, several other minor alleles of *Taq*IA and *Taq*IB are more frequent among OUD patients compared to healthy controls (353, 355, 356, 360, 351).

**Table 2:** Polymorphisms associated with OUD in the dopamine system and imaging correlates.

Gene	Polymorphism	Location	Findings	Author	Year	n	Ethnicity	Imaging Correlates
***DRD1***	**rs10078866**	Promoter	No significant association with OUD	Zhu et al. (346)	2013	939	Han Chinese	
				Liu et al. (53)	2013	739	Han Chinese	
	**rs10078714**	Promoter	No significant association with OUD	Liu et al. (53)	2013	739	Han Chinese	
	**rs1799914**	Exon 1	No significant association with OUD	Zhu et al. (346)	2013	939	Han Chinese	
	**rs265975**	3’ Near	Risk factor for OUD	*Jacobs et al. (347)	2014	286	Caucasian	
	**rs265973**	3’ Near	Risk factor for OUD	*Jacobs et al. (347)	2014	286	Caucasian	
	**rs686**	3’ UTR	Risk factor for OUD	Jacobs et al. (347)	2013	187	African American	
			No significant association with OUD	Zhu et al. (346)	2013	939	Han Chinese	
				Liu et al. (53)	2013	739	Han Chinese	
				Levran et al. (348)	2015	801	African American	
				Levran et al. (349)	2009	369	African American	
	**rs267418**	3’ UTR	No significant association with OUD	Peng et al. (350)	2013	739	Han Chinese	
	**rs6882300**	3’ UTR	No significant association with OUD	Peng et al. (350)	2013	739	Han Chinese	
	**rs2168631**	3’ UTR	No significant association with OUD	Peng et al. (350)	2013	739	Han Chinese	
	**rs5326**	5’ UTR	Risk factor for OUD	*Levran et al. (349)	2009	369	African American	
				Liu et al. (53)	2013	739	Han Chinese	
			No significant association with OUD	Zhu et al. (346)	2013	939	Han Chinese	
				Peng et al. (350)	2013	739	Han Chinese	
	**rs4532**	5’ UTR	No significant association with OUD	Zhu et al. (346)	2013	939	Han Chinese	
				Peng et al. (350)	2013	739	Han Chinese	
				Liu et al. (53)	2013	739	Han Chinese	
			No significant association with methadone dose	Crettol et al. (134)	2008	455	Caucasian	
	**rs4867798**	5’ UTR	No significant association with OUD	Zhu et al. (346)	2013	939	Han Chinese	
				Liu et al. (53)	2013	739	Han Chinese	
	**rs10063995**	5’ UTR	No significant association with OUD	Zhu et al. (346)	2013	939	Han Chinese	
	**rs265981**	5’ UTR	Protective against OUD	Liu et al. (53)	2013	739	Han Chinese	
***DRD2***	**rs6275**	Exon 7	Risk factor for OUD	Wang et al. (351)	2016	633	Han Chinese	
			Higher methadone dose	Doehring et al. (44)	2009	184	Caucasian	
			No significant association with OUD	Al-eitan et al. (352)	2012	425	Jordanian Arabic	
				Doehring et al. (44)	2009	184	Caucasian	
	**rs6277**	Exon 7	Higher response rates to methadone treatment	Crettol et al. (134)	2008	455	Caucasian	
			No significant association with OUD	Doehring et al. (44)	2009	184	Caucasian	
				Crettol et al. (134)	2008	455	Caucasian	
	**rs1801028**	Exon 7	No significant association with OUD	Doehring et al. (44)	2009	184	Caucasian	
	**rs1125394**	Intron 1	Risk factor for OUD	Wang et al. (351)	2016	633	Han Chinese	
				Al-eitan et al. (352)	2012	425	Jordanian Arabic	
	**rs17115583**	Intron 1	Protective against OUD	Wang et al. (351)	2016	633	Han Chinese	
	**rs1079597 (taqIB)**	Intron 1	Risk factor for OUD	Tsou et al. (353)	2017	950	Han Chinese	-Low D2R availability in healthy controls (334, 336*)
				*Zhang et al. (354)	2018	593	Han Chinese	
				Xu et al. (355)	2004	799	Chinese	
				Wang et al. (351)	2016	633	Han Chinese	
				Vereczkei et al. (337)	2013	858	Central European	
			No significant association with methadone dose	Huang et al. (272)	2016	138	Taiwanese	
	**rs4648319**	Intron 1	Risk factor for OUD	Tsou et al. (353)	2017	950	Han Chinese	
	**rs4648317**	Intron 1	No significant association with OUD	Doehring et al. (44)	2009	184	Caucasian	
	**rs7350522**	Intron 1	No significant association with OUD	Wang et al. (351)	2016	633	Han Chinese	
	**rs2075654**	Intron 2	Risk factor for OUD	Al-eitan et al. (352)	2012	425	Jordanian Arabic	
	**rs2734836**	Intron 2	Risk factor for OUD	Al-eitan et al. (352)	2012	425	Jordanian Arabic	
	**rs1800498 (taqID)**	Intron 2	Risk factor for OUD	Tsou et al. (353)	2017	950	Han Chinese	
				*Xu et al. (355)	2004	799	Chinese	
			No significant association with OUD	Vereczkei et al. (337)	2013	858	Central European	
				Doehring et al. (44)	2009	184	Caucasian	
				Xu et al. (355)	2004	663	German	
	**rs2283265**	Intron 4	Risk factor for OUD	Al-eitan et al. (352)	2012	425	Jordanian Arabic	
				*Levran et al. (348)	2015	801	African American	
			No significant association with OUD	Zhang et al. (354)	2018	593	Han Chinese	
	**rs1076560**	Intron 6	Risk factor for OUD	Al-eitan et al. (352)	2012	425	Jordanian Arabic	-Lower levels of striatal D2R availability in healthy controls (344) -No association with striatal D2R availability in healthy controls (345)
				Doehring et al. (44)	2009	184	Caucasian	
				Clarke et al. (341)	2014	2649	African American and European American	
				*Levran et al. (348)	2015	801	African American	
			No significant association with OUD	Zhang et al. (354)	2018	593	Han Chinese	
							
	**rs2734842**	3’ Near	Risk factor for OUD	*Zhang et al. (354)	2018	593	Han Chinese	
	**rs2242591**	3’ Flanking Region	Risk factor for OUD	*Zhang et al. (354)	2018	593	Han Chinese	
	**rs6278**	3’ UTR	Risk factor for OUD	*Zhang et al. (354)	2018	593	Han Chinese	
	**rs6279**	3’ UTR	Risk factor for OUD	*Zhang et al. (354)	2018	593	Han Chinese	
	**rs1799732**	5’- UTR	Risk factor for OUD (C deletion)	Al-eitan et al. (352)	2012	425	Jordanian Arabic	-Combined minor variants associated with higher striatal D2R availability in healthy controls (334) -No association with extrastriatal D2R in healthy controls (340)
			No significant association with OUD	Teh et al. (356)	2012	93	Han Chinese	
				Zhang et al. (354)	2018	593	Han Chinese	
				Doehring et al. (44)	2009	184	Caucasian	
							
	**rs12364283**	5’ UTR	No significant association with OUD	Doehring et al. (44)	2009	184	Caucasian	
	**rs1799978**	5’ UTR	No significant association with OUD	Doehring et al. (44)	2009	184	Caucasian	
				Teh et al. (41)	2012	93	Han Chinese	
			Risk factor for OUD	*Hung et al. (357)	2011	321	Han Chinese	
			Higher methadone doses	Hung et al. (357)	2011	321	Han Chinese	
			No significant association with relapse rates on methadone treatment	Bawor et al. (358)	2015	240	Mixed	
				Doehring et al. (44)	2009	184	Caucasian	
***ANKK1***	**rs4938013**	Exon 2	Risk factor for OUD	Nelson et al. (359)	2013	3485	Caucasian	
				*Zhang et al. (354)	2018	593	Han Chinese	
	**rs7118900**	Exon 5	Risk factor for OUD	*Zhang et al. (354)	2018	593	Han Chinese	
				*Levran et al. (348)	2015	801	African American	
	**rs1800497 (taqIA)**	Exon 8	Risk factor for OUD	Teh et al. (356)	2012	93	Han Chinese	-Low D2R availability in healthy controls (334, 335, 336*)
				Hou and Li (360)	2009	1030	Chinese/East Asian	
				*Vereczkei et al. (337)	2013	858	Central European	
				Tsou et al. (353)	2017	950	Han Chinese	
				*Zhang et al. (354)	2018	593	Han Chinese	
				*Doehring et al. (44)	2009	184	Caucasian	
			No significant association with OUD	Al-eitan et al. (352)	2012	425	Jordanian Arabic	
				Barratt et al. (361)	2006	166	Mixed	
			No significant association with methadone dose	Crettol et al. (134)	2008	455	Caucasian	
			No significant association with methadone or buprenorphine therapy success	Barratt et al. (361)	2006	166	Mixed	
			Improved withdrawal among methadone-maintained patients	Barratt et al. (361)	2006	166	Mixed	
	**rs877138**	5’- Flanking Region	Risk factor for OUD	Nelson et al. (359)	2013	3485	Caucasian	
***DAT1***	**9-repeat VNTR**	3’ UTR	Risk factor for OUD	Galeeva et al. (362)	2002	287	Caucasian males	-Higher striatal DAT availability (363–365) -No association with striatal DAT availability (366, 367)
			No significant association with OUD	Hou and Li (360)	2009	1030	Han Chinese	
				Yeh et al. (368)	2010	1046	Han Chinese	
							
	**10-repeat VNTR**	3’ UTR	Risk factor for OUD	Ornoy et al. (369)	2016	158	Israeli Jewish Females	-Higher striatal DAT availability (370, 371) -No association with striatal DAT availability (366, 367)
							

SNP associations refer to the minor allele.

*Nominal significance.

There is less robust evidence for other *DRD2* polymorphisms in OUD. For example, despite preclinical evidence that rs1076560 may alter D2R expression, genetic association studies between rs1076560 and OUD have been inconsistent (44, 341, 348, 352, 354). In contrast, while the role of rs1799732 on D2R expression is uncertain, subjects with the minor variant have shown to be at higher risk for OUD in the Jordanian Arabic population (352).

The extent to which *DRD2* polymorphisms affect the response to MOUD in patients with OUD is inconsistent across studies. Lawford et al. (372) first reported that the *Taq*IA1 allele was associated with poorer treatment outcomes among Caucasian patients on methadone maintenance therapy. Since then, no group has replicated these findings in Caucasian populations (44, 134, 358, 361). Similarly, no association was found between *Taq*IB and methadone maintenance therapy response nor *Taq*IA and buprenorphine maintenance therapy response (44, 272). However, Crettol et al. (134) did report an association with rs6277 and patients’ response to methadone maintenance therapy; patients with the major CC genotype were more likely to abuse illicit opioids on methadone therapy than those with CT or TT genotype. Interestingly, in two [^11^C]raclopride PET studies, the major C allele of rs6277 was associated with lower striatal D2R availability in healthy volunteers (373, 374), while another [^11^C]FLB457 PET study found the C allele predicted high extrastriatal D2R availability across the cortex and hypothalamus (340). However, several studies found no association between rs6277 and OUD (44, 134). Further, Doehring et al. (44) found no relationship between rs6277 and methadone maintenance therapy response. Instead, this group found that minor allele carriers of a different polymorphism, rs6275, required greater methadone doses than non-carriers and took longer to reach their maximum methadone dose (44). Thus, genetic studies suggest a role of *DRD2* polymorphisms in treatment response in OUD; however, they remain inconsistent and difficult to replicate.

Several studies have investigated the role of *DRD2* variants on behaviors associated with OUD. The tridimensional personality questionnaire scores personality on harm avoidance, novelty seeking, and reward dependence (375). These scores are used to calculate a borderline index using the equation: borderline index = harm avoidance + novelty seeking − reward dependence (376). Borderline index reflects borderline personality trait, characterized by a fear of abandonment, self-injurious behaviors, and emotional dysregulation (376, 377) (DSM-5). A recent study found that OUD patients had higher harm avoidance and novelty seeking scores and lower reward dependence scores, and thus a higher borderline index, than healthy volunteers (356). Further, Huang et al. (272) found that borderline index scores are inversely correlated with methadone dose, indicating the relevance of borderline index score in OUD treatment. These personality scores have not shown associations with *Taq*IA or *Taq*IB polymorphisms (272, 356). However, the -141C *Del* polymorphism (rs1799732) is associated with higher harm avoidance scores among OUD patients (356). In contrast, Gerra et al. (377) found that OUD patients had lower harm avoidance scores compared to CUD patients and healthy volunteers. However, this study reported that both CUD and OUD patients had higher novelty seeking scores and lower reward dependence scores than healthy volunteers (378). Therefore, this difference in harm avoidance could be rooted in genetic differences between the groups, as -141C *Del* is associated with higher harm avoidance scores in OUD, though Gerra et al. (378) did not report the genetic composition of their cohort (356).

#### Molecular Imaging: D2R and OUD

[^11^C]raclopride and [^123^I]IBZM are widely used radiolabeled D2R antagonists differing *via* regioselectivity used to study D2R distribution, with additional affinity to D3Rs (D2-like inhibitory receptors) (379–381). [^11^C]NMB is another radiotracer used to study D2R availability with higher affinity for D2Rs over D3Rs than [^11^C]raclopride and [^123^I]IBZM (382, 383). Lastly, [^11^C]FLB-457 is a high-affinity radioligand that targets extrastriatal D2Rs and D3Rs (384).

In contrast to other SUDs, less is certain about D2R availability in OUD. In one [^11^C]raclopride PET study, OUD participants showed lower D2R availability compared to healthy controls (6). In this study, all OUD patients actively used heroin and most, but not all, were also on methadone therapy (6). In another [^11^C]raclopride PET study, recently detoxed OUD patients showed lower D2R availability than healthy controls (7). These patients also demonstrated lower levels of DA release in response to a methylphenidate challenge in comparison to healthy controls (7). In a [^123^I]IBZM SPECT study, the OUD patients were abstinent without maintenance therapy anywhere from 1 to 24 weeks (318). Zijlstra et al. (318) observed a negative correlation in length of opioid use history with striatal D2R availability. In contrast, two [^11^C]raclopride studies observed no differences in D2R availability between OUD patients receiving methadone therapy and healthy controls (19, 20). These findings suggest the potential therapeutic benefit of MOUD in restoring neurochemical imbalances resulting from substance abuse. These results demand further investigation into the relationship between OUD and D2R availability, particularly in the context of MOUD.

### DRD1

#### *DRD1* Background

The D1R is the most abundant DA receptor in the brain (380). Coded by *DRD1*, this excitatory GPCR is widespread, but most densely expressed in the dorsal striatum, hippocampus, amygdala, and neocortex, as illustrated in **Figure 1** (385–388). D1Rs influence learning and memory *via* association with N-methyl-D-aspartate (NMDA)-mediated long-term potentiation as well as impact D2R-mediated events and regulate addiction-associated behaviors such as impulsivity (389–396). D1Rs are important mediators of several reward-related processes and there is evidence that D1Rs are required and sufficient for drug reward and conditioning (397, 398).

D1R function is relevant in OUD because DA release triggered by opioid-induced MOP receptor activation indirectly stimulates D1Rs and associated reward circuitry (69). While one post-mortem study showed lower D1R mRNA levels in the putamen and NAc shell in OUD subjects relative to controls (347), another postmortem analysis showed higher D1R mRNA and protein expression in VTA, NAc, and amygdala in the brains of opioid abusers compared to controls (399). This difference may be attributed to the difference in populations studied. Where Sadat-Shirazi et al. (399) studied patients who exclusively abused opioids, Jacobs et al. (347) included polysubstance users.

In addition, pharmacological manipulations of D1Rs in preclinical models of OUD demonstrate alterations in behaviors associated with dependence and withdrawal. For example, infusion of D1R agonist SKF 38393 into the NAc enhances, while antagonist SCH 23390 blunts, conditioned place preference in morphine-addicted rats (400). Additionally, infusions of SCH 23390 into the NAc core reduced cue-induced heroin-seeking in dependent rats (401). Furthermore, D1R agonist SKF 82958 relieved naloxone-precipitated withdrawal symptoms in morphine-dependent rats (402). These findings highlight the importance of D1Rs in OUD and correspond with other SUD models. For example, SCH 23390 infusion blocks reinstatement of cocaine-seeking in rats, while D1R agonist SKF 81297 reinstates cocaine-seeking (403, 404). In addition to pharmacological D1R blockade, D1R knock-out mice fail to self-administer cocaine (397). In models of AUD, NAc shell infusions of SCH 23390 blunt, while infusions of D1R agonist A-77636 enhance, ethanol self-administration in alcohol-preferring rats (405).

D1 and MOP receptors directly colocalize into hetero-oligomers in the rat cortex and striatum (including accumbens nucleus), regions of importance in reward and locomotor activity. Together, they promote locomotor sensitization in rats chronically treated with morphine, suggesting this association may be involved in the long-term neuronal changes associated with addiction (406, 407).

#### *DRD1* Polymorphisms

While less attention has been given to variations in *DRD1* than *DRD2/ANKK1*, there are several functional polymorphisms that have been studied in the context of SUDs. One study demonstrated that rs5326A, located in the 5’ untranslated region, correlated with decreased *DRD1* promoter strength in an *in vitro* luciferase model (408). Other *DRD1* polymorphisms may increase vulnerability to OUD by interacting with the glutamatergic system in the brain. Homer scaffold protein 1 (*HOMER1*) encodes HOMER1, a postsynaptic protein that facilitates glutamatergic transmission (409). Excitatory glutamatergic signaling has been shown to underlie the persistent compulsion to use drugs, suggesting SNPs disrupting this gene interaction may be relevant in OUD (410). In a post-mortem analysis of Caucasian samples, the *DRD1* polymorphism rs265973 associated with HOMER1 expression in the striatum (347). Interestingly, the minor T allele associated with higher levels of striatal HOMER1 mRNA among the OUD cohort, but associated with lower levels of striatal HOMER1 mRNA in the control cohort (347). Thus, it is possible *HOMER1*-associated genetic variants disrupt glutamatergic and dopaminergic signaling and contribute to OUD.

#### Genetic Association Studies: *DRD1* and OUD

Preliminary findings suggest a role of *DRD1* SNPs in OUD, as outlined in **Table 2**. For example, Liu et al. (411) found that two SNPs located in the 5’ untranslated region of *DRD1*, major allele rs265981G and minor allele rs5326A, associated with OUD in a Han Chinese population. Levran et al. (348, 349) also found a trend toward an association between rs5326A and OUD in an African American sample. However, other groups were unable to replicate these findings (346, 350). Jacobs et al. (347) found a nominally significant association between *DRD1* SNP rs265973 and OUD among Caucasians, but not African Americans. This provides further evidence of an association between *HOMER1* and OUD, perhaps with ethnicity-dependent effects.

Several studies demonstrate that *DRD1* variants associate with the duration of transition from the first use to dependence of opioids (346, 350). The duration of transition from the first use to dependence is of clinical significance; patients with a more rapid transition to dependence have poorer treatment outcomes and more severe SUDs (412, 413). Zhu et al. (346) found that the minor alleles of rs686 and rs4532 associated with a longer transition period. Peng et al. (350) were unable to replicate the rs4532 association, but found that homozygotes for the major alleles of rs5326 and rs6882300 had an accelerated transition to OUD. Interestingly, while these SNPS associated with the transition from first use to dependence, neither study found that they were associated with increased risk for OUD (346, 350).

*DRD1* variants have also been implicated in subjective ratings of pleasure in response to opioids, both upon first use and after dependence (346). Typically, the pleasurable feeling associated with opioids increases with duration of use: most patients report a negative response upon their first use and a euphoric response after dependence (133, 346). This suggests that chronic opioid use induces changes to reward-related circuitry. One potential mechanism is through D1R-mediated phosphorylation of NMDA, contributing to long-term potentiation (414). *DRD1* variants have been associated with this reward sensitization process in a Han Chinese population (346). This study revealed that *DRD1* SNPs that modulate the subjective response to opioids upon first use are distinct from those that do so after dependence. Specifically, the minor alleles of rs5326, rs10063995, and rs10078866 are associated with a non-pleasurable first use of opioids, but are not associated with the subjective response after dependence. Conversely, the minor variants of rs686 and rs4532 are associated with less pleasurable responses to opioids after dependence, but are not associated with the initial response (346). Findings from a rat study indicate that there is a reward-switching mechanism in opioid response within the basolateral amygdala in which D1R signaling is associated with reward upon first use and D2R signaling with reward after dependence (415). Thus, it is possible that rs686 and rs4532 associate with less pleasurable opioid responses after dependence by modulating D2R activity.

#### Molecular Imaging: D1R and OUD

No molecular imaging studies have yet assessed D1R availability in OUD or in *DRD1* polymorphism carriers. Few studies have examined the relationship between other SUDs and D1R levels. [^11^C]NNC 112 and [^11^C]SCH 23390 are radiolabeled D1R antagonists that differentially distribute throughout the brain; however, both display high affinity in the striatum and extrastriatal regions (416–418). In one [^11^C]NNC 112 study, D1R availability in CUD patients was not significantly different than in healthy controls (419). In contrast, studies utilizing [^11^C]SCH 23390 PET reveal individuals with tobacco use disorder have lower D1R availability than healthy controls (420, 421). These limited findings highlight the need for increased investigation into D1R availability in addiction.

### DAT1

#### *DAT1* Background

DAT are plasma membrane proteins essential for the clearance of DA from the synapse; they play a critical role in regulating DA neurotransmission, especially in the striatum (422–426). DAT harness the electrochemical gradient to transport two sodium ions with a DA molecule into the cell, thus regulating extracellular DA concentrations (423). DAT are coded by *DAT1*, a gene widely studied for its role in substance abuse (427).

#### *DAT1* Polymorphisms

The most studied polymorphisms of *DAT1* are VNTRs in the 3’ untranslated region, which may affect DAT expression (428–431). The most common variants are those with 9 or 10 repeats of the 40 base pair sequence (432) and multiple molecular imaging studies have investigated their functional effects. In several [^123^I]β-CIT SPECT studies, 9-repeat VTNR carriers demonstrated higher striatal DAT availability than the 10-repeat homozygotes (363–365). In contrast, two [^123^I]β-CIT SPECT studies found those homozygous for the 10-repeat allele had higher striatal DAT density compared to non-10-repeat carriers (370, 371). Finally, Martinez et al. (366) and Lynch et al. (367) found no effect of VNTR polymorphisms on striatal DAT expression in a [^123^I]β-CIT SPECT and [^99m^Tc]TRODAT-1 study, respectively. Lastly, Guindalini et al. (433) found that the rare 6-repeat VNTR genotype reduced DAT1 expression *in vitro*, particularly when cocaine was added to the culture. However, the effects of the 6-repeat VNTR polymorphism on *DAT1* availability has not been assessed *in vivo* with PET methodology. Thus, further research is required to determine these polymorphisms’ functional effects on DAT expression and availability.

#### Genetic Association Studies: *DAT1* and OUD

Genetic association studies of *DAT1* and OUD have yielded inconsistent results. While Galeeva et al. (362) found an association between 9-repeat VNTR allele and OUD in an ethnic Russian and Tartar male population, later studies in Han Chinese populations did not observe any association (360, 368). Ornoy et al. (369) examined the heritability of *DAT1* ADHD risk alleles in Sephardic and Ashkenazi Jewish heroin-dependent individuals and their children. They found that mothers with OUD were more likely to be carriers of the *DAT1* 10-repeat allele than mothers without OUD. This association was not seen in fathers and was not explained by prevalence of ADHD among mothers with the polymorphism. Further, the children of heroin-dependent parents were more likely to inherit the 10-repeat allele than children of healthy volunteers (369). However, it is unclear how these VNTR polymorphisms impact DAT availability and thus vulnerability to OUD, as molecular imaging studies have conflicting results (363–367, 370, 371).

Polymorphisms in *DAT1* have been associated with other SUDs, which may provide insight into their functional effects on DA signaling in addiction. *DAT1* VNTR has been associated with OUD (362) as well as AUD in Western European and Japanese populations (47, 434). A meta-analysis also found that the 9-repeat VNTR was associated with increased withdrawal severity in AUD (435). The 6-repeat VNTR genotype was found to be a risk factor for CUD, but this variant has not yet been studied in OUD (45). Thus, it seems that *DAT1* VNTR polymorphisms may affect DAT expression and contribute to SUDs.

Evidence suggests that the number of VNTR in patients with OUD influences their response to treatment. In each study, a “poor” treatment outcome indicates continued heroin use or treatment drop-out, whereas a “successful” outcome indicates cessation of illicit opioid use. In patients receiving buprenorphine therapy, carriers of the 10-repeat VNTR allele had poor outcomes more often than successful outcomes (436). Conversely, 6-, 7-, and 11-repeat VNTR allele carriers had successful outcomes in response to buprenorphine therapy more often than not (436). Gerra et al. (436) suggest that these variations in *DAT1* may modulate buprenorphine-associated DA transmission and thus affect treatment success. In a study of both oral and implanted naltrexone therapy, Krupitsky et al. (437) found that OUD patients with the 9-repeat VNTR allele had poor outcomes more often than successful ones on both forms of naltrexone. Thus, genotyping *DAT1* VNTR could be useful in OUD therapy selection.

While van Gestel et al. (438) reported an association between *DAT1* VNTR polymorphisms and novelty seeking, a personality trait associated with SUDs (439), other studies have failed to replicate this finding (440, 441).

#### Molecular Imaging: DAT and OUD

Several molecular imaging studies have assessed DAT availability in SUDs utilizing DAT-sensitive tracers including [^99m^Tc]TRODAT-1, [^123^I]β-CIT, [11C]WIN 35,428, [11C]cocaine, and [^11^C]CFT. There is evidence from molecular imaging studies that DAT availability is altered in SUDs. For example, CUD is associated with higher striatal DAT concentrations compared to healthy controls (54, 55), while methamphetamine-dependent individuals demonstrate lower striatal DAT availability compared to healthy controls (51, 57, 58). Alcohol and tobacco dependence have also been associated with lower striatal DAT levels (59, 60–62); however, other studies have observed no association between DAT levels and alcohol and tobacco dependency (22, 442). Although varied, these results overall suggest that DAT plays a role in SUDs.

PET and SPECT studies suggest that OUD is associated with decreased DAT availability. Chronic heroin users, detoxed abstainers, and methadone-maintained patients all present lower striatal DAT levels than healthy controls (48–53). A [^99m^Tc]TRODAT-1 SPECT study comparing DAT concentrations between recently detoxed heroin-dependent patients and recently detoxed methamphetamine-dependent patients showed that both had lower striatal DAT availability than healthy controls and had no differences between them (51). In contrast, Cosgrove et al. (443) utilizing [^123^I]β-CIT SPECT imaging, reported no differences in striatal DAT levels between heroin users and healthy controls, though they acknowledged the limitations of their small sample sizes (443).

DAT availability may also vary based on the use of MOUD. For example, one [^11^C]CFT PET study reported methadone-maintained OUD patients showed lower DAT availability in the bilateral putamen than abstinent OUD patients, with both presenting lower striatal DAT availability compared to healthy controls (49). Further, while methadone-maintained patients showed lower DAT availability in caudate and putamen compared to controls, abstinent OUD patients showed lower DAT availability in the caudate only, suggesting that abstinence from opioids may partially recover DAT availability (49). However, a [^99m^Tc]TRODAT-1 SPECT study found similar striatal DAT availability between methadone-maintained and abstinent OUD patients (50). This discrepancy may be due to methodological differences; in one study, patients were at least 6 months abstinent (49), while in the other, patients were abstinent for only 3 months or less (50). In a within-subjects [^99m^Tc]TRODAT-1 SPECT study, Liu et al. (53) observed a 14–17% increase in DAT levels in the caudate and putamen of 64 heroin-dependent patients after 6 months of treatment with traditional Chinese Jitai tablets, an herbal remedy associated with withdrawal mitigation. No significant increase in DAT levels was observed in the placebo-treated group. However, even among the medication group, DAT availability was not restored to that of healthy control levels (53). Thus, further studies are required to determine the effects of MOUD compared to sustained abstinence on DAT availability.

## Conclusion

Preclinical and clinical studies have demonstrated the importance of the opioid and DA systems in SUDs, including OUD. Polymorphisms within these systems have functional consequences that may influence a number of modalities in addiction, including vulnerabilities, addiction severity, treatment response, and relapse rates. PET and SPECT methodology allow for the study of these receptor systems in both healthy and substance-dependent populations and provide insight into the neurobiology of OUD.

Within the opioid system, the MOP receptor has been most closely studied in the context of OUD. The minor allele of the *OPRM1* rs1799971 SNP has been widely linked to a reduction in MOP receptor availability (100–105). The implications of this in OUD, however, remain elusive; findings from genetic association studies are varied and seem largely ethnicity-dependent (109). The KOP and NOP receptors have also been studied in relation to OUD; both play important roles in the dysphoric effects of drug abuse seen during withdrawal, including modulating activation of the HPA axis (16, 291, 294, 295, 444). A number of polymorphisms in *OPRK1* have been associated with OUD and opioid withdrawal severity (147, 155, 156, 232). Similarly, VNTR polymorphisms in *PDYN* have been correlated with opioid withdrawal, suggesting the importance of dynorphin-KOP receptor signaling system in the mediation of stress-induced withdrawal and compulsive drug-seeking (155). Lastly, genetic variants in both *PDYN* and *OPRL1* have been associated with personality traits and behaviors associated with SUDs, another indication of their roles in OUD (235, 236, 445). The DOP receptor has an inverse function to the KOP receptor, in that DOP receptor activation improves negative emotional states (255). While several *OPRD1* polymorphisms correlated with heroin dependence (130, 136, 138–141, 146, 150), it is likely that the effects are ethnicity-dependent, as several other studies found no significant associations between *OPRD1* polymorphisms and OUD (137, 140, 144).

The DA system has several well-studied polymorphisms that have been linked with OUD and other SUDs. For example, polymorphisms in *DRD2/ANKK1*, in particular the *Taq*IA and *Taq*IB SNPs, may result in lower D2R availability (321, 334, 335) and have been associated with addictive behaviors including OUD (330, 332, 354, 446, 447). Less studied *DRD2* polymorphisms may also affect D2R expression (341, 344) but results have been varied. Additionally, *DRD2* polymorphisms may associate with response to medications for OUD; however, there are conflicting reports and further research is required (44, 134, 272, 335, 358, 361, 372). Fewer conclusions can be drawn about *DRD1*; for example, several *DRD1* polymorphisms were initially associated with a rapid transition from first opioid use to opioid dependence, but the results could not be replicated (346, 350). Lastly, lower DAT availability has also been associated with OUD (48–53). Both the 9- and 10-repeat VNTR alleles have been associated with lower DAT availability (363–365, 370, 351); thus, more studies are required to pinpoint the effects of the different repeat VNTR polymorphisms in OUD.

While there is strong preliminary evidence of the role of genetic variants in the DA and opioid systems in OUD, more molecular imaging studies are required in individuals with OUD. In particular, studies utilizing PET tracers that target the less-studied opioid receptors, D1R, D3R, and DAT, would greatly contribute to our understanding of the complex interplay between these receptors in opioid addiction. For instance, as of yet, no imaging studies have examined DOP, KOP, NOP, D1, or D3 receptors in individuals with OUD. One of the most important molecular imaging research questions in OUD is how the different MOUD may alter the dopamine and opioid receptor systems and if these changes are associated with higher rates of successful abstinence. Current imaging studies largely group abstinent and medication-maintained OUD participants together and compare to healthy controls; however, analyses between OUD subgroups would shed light on any neurochemical benefits of MOUD. This would help inform treatment and ultimately improve outcomes for those suffering from OUD. Additionally, opioid receptor antagonist challenge studies would help assess the interaction between drugs like naloxone and semi-synthetic or synthetic opioids, improving safety and efficacy of overdose reversal and prevention. Finally, molecular imaging studies examining the effects of polymorphisms in the DA and opioid systems would help elucidate the genetic components of OUD. The literature relating to genetic association studies in OUD does suggest that certain polymorphisms are risk factors for OUD or may affect treatment outcomes. However, given that these associations are largely ethnicity-dependent, it is important to replicate these findings. Finally, there seem to be sex effects both on genetic association studies and PET/SPECT findings; therefore, future studies could investigate the sex differences in development and outcome of OUD. Further investigation into the underlying genetic factors of OUD and treatment response is critical to help curb the opioid crisis by means of addiction prevention, novel pharmacological targets, and precision treatment.

## Author Contributions

PM, CW, NV, and G-JW contributed to the conception and design of the study. JB, DK, DF, and CK conducted a literature search and wrote the first draft. All authors contributed to manuscript revision, and read and approved the submitted version.

## Conflict of Interest Statement

The authors declare that the research was conducted in the absence of any commercial or financial relationships that could be construed as a potential conflict of interest.
